# The buffering effect of spousal support in later life: mediated and moderated pathways from negative life events to loneliness among older adults

**DOI:** 10.3389/fpsyg.2025.1698354

**Published:** 2025-11-14

**Authors:** Guangjie Yuan, Zihan Cheng, Jiaojian Wang, Lin Wang

**Affiliations:** 1 Faculty of Education, Qufu Normal University, Qufu, China; 2 College of Psychology, Qufu Normal University, Qufu, China

**Keywords:** loneliness, negative life events, marital satisfaction, spousal support, older adults

## Abstract

**Objectives:**

Based on stress response theory and the social ecological model, this study systematically examines the mechanisms underlying the impact of negative life events (NLEs) on loneliness among older adults, with a focus on the mediating role of marital satisfaction (MS) and the moderating effects of spousal support.

**Methods:**

Employing a cross-sectional design, we administered standardized assessment tools—including the UCLA Loneliness Scale (Version 3), NLEs Inventory, ENRICH Marital Satisfaction Brief Scale, and Spousal Support Scale—to a national convenience-sample of individuals aged 60 years and above, yielding 469 valid participants.

**Results:**

Results indicate that NLEs exert both direct and indirect effects on loneliness, with the latter operating via reduced MS. Notably, spousal support demonstrates dual moderating effects: Low levels of support amplify the detrimental impact of adversity on marital quality and thereby intensify loneliness; whereas high levels counteract these negative pathways and foster relational resilience.

**Discussion:**

This study provides the first empirical evidence for the dual-directional moderating role of spousal support, which not only buffers against stress but also cultivates relational adaptability. Findings offer critical theoretical insights for developing family-centered mental health interventions tailored to older adults.

## 1 Introduction

China is undergoing the most rapid and extensive population aging process in human history. According to the latest statistics from the National Health Commission, China’s population aged 60 and above has reached 310 million, accounting for 22% of the total populace. Projections by the National Bureau of Statistics indicate that by 2035, this demographic will surpass 400 million, constituting over 30% of the national population—a threshold marking China’s formal entry into super-aged society. Against this backdrop, mental health among older adults has evolved from an individual concern to a priority within national public health agendas.

Compared to other age cohorts, older adults confront multifaceted challenges, including physiological decline, diminished social roles, and weakened intergenerational support systems (Theeke, 2009). These risk factors impair the social adaptability of older adults, thereby markedly elevating their susceptibility to loneliness (Zhu, 2023). Data from the China Health and Retirement Longitudinal Study (CHARLS) reveals that 28.3% of individuals aged 65 and above experience significant loneliness (Zhang and Li, 2025). As a cardinal indicator of subjective social isolation, loneliness is empirically linked to adverse health outcomes such as cognitive impairment, depression, and functional decline (Zhang and Li, 2025), imposing direct annual healthcare costs amounting to approximately RMB 32.7 billion (National Medical Insurance Administration, 2025). Consequently, elucidating the determinants and mechanisms underlying loneliness in later life holds critical significance for advancing theoretical understanding of geriatric mental health and informing targeted policy interventions to enhance quality of life among older populations.

Drawing upon the social ecological model (Bronfenbrenner, 2000), we hypothesize that NLEs exacerbate loneliness through dual pathways: directly related to loneliness (Tang et al., 2024), and indirectly amplifying isolation via disruptions to marital relationships—a cornerstone of late-life support networks (Ralston et al., 2022). The model emphasizes the interaction between individuals and multi-level environmental systems (e.g., family, community), providing a systematic theoretical perspective for this study to examine the role of MS and spousal support in the “life events–loneliness” pathway. MS serves as a linchpin in this dynamic; its deterioration weakens the “psychological safe harbor” function of spousal partnerships, precipitating crises of perceived irrelevance among older adults (Tang et al., 2024). Notably, spousal support acts as a pivotal moderator: high-quality support facilitates adaptive coping, whereby adversities strengthen emotional bonds through empathy and collaboration (Koranyi et al., 2017). Conversely, low support accelerates marital dissolution, perpetuating a vicious cycle of “event → dissatisfaction → loneliness” (Aguilar-Raab et al., 2022). A comparative study of Spanish and Dutch samples found that the quality—rather than the quantity—of social support exerts a stronger influence on explaining loneliness among older adults (Rodrigues et al., 2014).

This study adopts a framework that combines the social ecological model and stress response theory to dissect the mediating pathways linking NLEs to loneliness in older adulthood. Integrating the social-ecological model with stress response theory, this study systematically examines the influence pathways through which negative life events influence loneliness among older adults. Stress response theory emphasizes the psychological and physiological adaptation processes under pressure, helping to explain how adverse events may deplete psychological resources, trigger emotional dysregulation, and consequently affect marital quality and the experience of loneliness. It prioritizes examining the mediating role of MS and the contextual moderation effects of spousal support. Our research employs a multidimensional analytical approach to elucidate the differential mechanisms underlying adverse event effects. Findings will not only provide empirical validation and expansion of stress response and ecological theories but also deepen theoretical insights into the etiology of late-life loneliness.

### 1.1 Conceptualization of the relationship between NLEs and loneliness in older adults

The NLEs refer to stressful changes or sudden occurrences with pronounced adverse effects that individuals encounter (Rabkin and Struening, 1976). These events encompass a broad range, including deteriorating health conditions, loss of close relationships, financial hardship, escalating family conflicts, and fractured social ties (Mo et al., 2020). These events are defined by their uncontrollability and prolonged impact, which collectively pose profound threats to both psychological and physiological well-being in older adulthood. Extensive research demonstrates that NLEs constitute a significant risk factor for depressive symptoms and may even precipitate suicidal behavior (Cacioppo et al., 2010). Notably among older adults facing critical life transitions such as terminal illness diagnoses or spousal bereavement, levels of depressive affect and suicide risk exhibit marked increases (Chang et al., 2010). Moreover, NLEs correlate with cognitive decline, physical frailty, and increased mortality rates, thereby systematically eroding overall well-being and quality of life in later life (Wei et al., 2022). The underlying pathogenic mechanism involves chronic stress responses elicited by NLEs, which progressively erode coping resources and fracture social support networks, ultimately accelerating health deterioration.

Empirical evidence consistently confirms a significant positive association between NLEs and loneliness in older adulthood (Aartsen and Jylhä, 2011; Chang et al., 2010). NLEs—including functional disability onset, loss of core family members, or adult children’s geographic relocation—directly elevate loneliness by weakening social connectedness and intensifying perceived isolation (Aartsen and Jylhä, 2011; Li et al., 2024). Cross-cultural studies corroborate these findings, identifying NLEs as a robust correlate of loneliness, with stronger associations emerging in resource-constrained communities (Aartsen and Jylhä, 2011; Wei et al., 2022).

Given this convergent evidence, we posit that NLEs exert a significant influence on loneliness in older adults. Accordingly, we propose the following hypothesis: NLEs significantly and positively predict levels of loneliness among older adults (H1).

### 1.2 The mediating role of MS between NLEs and loneliness in older adults

MS, as a central indicator of marital quality, encapsulates individuals’ global perceptions, emotional appraisals, and perceived fulfillment within their marital relationships (Olson, 2000). Substantial evidence confirms a significant negative correlation between NLEs and MS (Nguyen et al., 2021; Stith et al., 2008). When individuals or families encounter major life disruptions, these stressors impact the marital system through two distinct pathways. First, external pressures deplete psychological resources, leading to disrupted spousal interactions marked by heightened conflict and reduced positivity. This cascade erodes the relational foundation (Dion et al., 2023). Second, attributions made by partners regarding adverse events—whether blaming one another or framing challenges as shared burdens—significantly regulate the actual impact of these events on MS (Fincham and Bradbury, 1993).

Loneliness, defined as a distressing subjective experience stemming from discrepancies between actual and desired social interactions, is particularly prevalent among older adults (Theeke, 2009). Empirical findings demonstrate a robust positive association between MS and both mental health and psychological security in later life (Tang et al., 2024). High-quality marriages provide sustained emotional sustenance, profound validation, and daily companionship, effectively cushioning against isolation triggered by age-related transitions such as retirement, empty-nest syndrome, and bereavement; conversely, low-quality marital relationships—even when cohabitation persists—may exacerbate loneliness due to deficits in communication and emotional connectivity. For older adults, marital bonds represent the most stable and central component of their social support networks, rendering MS critically influential in mental health, particularly loneliness (Ralston et al., 2022).

Building upon this theoretical framework and empirical basis, we propose that NLEs not only directly undermine marital quality but may also indirectly exacerbate loneliness through the mediating role of MS. Although prior research has separately documented the erosive effects of NLEs on MS and the protective effects of MS against loneliness, the dynamic interplay between these three constructs remains underexplored. The unique vulnerability of marital systems in old age—exacerbated by physiological decline and shifting social roles—heightens susceptibility to disequilibrium following adverse events. Such disruptions may simultaneously disrupt marital equilibrium and weaken conjugal buffering against loneliness through declining MS. Consequently, we advance our second hypothesis: MS partially mediates the relationship between NLEs and loneliness in older adults (H2). Specifically, negative events directly shape marital evaluations while simultaneously undermining the marital relationship (a primary source of social support) thereby amplifying perceptions of social isolation and loneliness.

### 1.3 The moderating role of spousal support between NLEs and loneliness in older adults

Spousal support, a cornerstone of marital dynamics, encompasses multidimensional manifestations including emotional support (e.g., active listening and deep empathy), instrumental assistance (e.g., practical problem-solving), informational guidance (e.g., constructive advice), and respect-based affirmation (e.g., enhancing self-efficacy), collectively addressing spouses’ psychological and material needs (Lawrence et al., 2008). For older adults, spousal support holds particular significance—given that partners typically serve as the primary social support network, especially amid physical limitations (Lee and Chopik, 2025). Research demonstrates that high-quality spousal support substantially improves quality of life, reduces loneliness and depression risks, and possesses irreplaceable value amid age-related social network contraction (Lee and Chopik, 2025).

Negative life events (e.g., chronic illness, bereavement) may exacerbate loneliness through pathways including restricted social engagement or heightened psychological distress, yet spousal support functions as a critical buffer against these effects (Switsers et al., 2021; Tang et al., 2024). Empirical evidence reveals that robust spousal support mitigates negative interaction cycles triggered by stressors. When partners respond with empathy (e.g., perspective-taking, emotional validation) during conflicts, MS remains resilient despite adverse events (Marini et al., 2020). Similarly, collaborative support behaviors (e.g., joint problem-solving) fortify the marital alliance, counteracting loneliness’s erosive impact on the relationship (Koranyi et al., 2017). Further studies indicate that among couples with higher marital quality, cooperative coping strategies significantly alleviate the depletion of MS induced by negative events (Wilson et al., 2021); conversely, unsupported partnerships are more vulnerable to dissatisfaction following adversities (Choi and Ha, 2011).

While existing research comprehensively documents the dual threats of NLEs to loneliness and MS in later life, and corroborates spousal support’s central role in sustaining marital quality and mental health, an integrated examination of their interrelations remains underdeveloped. Although theoretical frameworks position spousal support as a protective factor against negativity, its differential moderating mechanisms across distinct pathways warrant systematic exploration. Accordingly, this study advances two hypotheses: H3: Spousal support moderates the association between NLEs and loneliness, such that high-level support weakens the association between adverse events and perceived social isolation or loneliness; H4: Spousal support moderates the impact of NLEs on MS, whereby supportive partner interactions mitigate the erosive effects of stressors on marital quality. This dual-moderation model aligns with stress-buffering theory’s conceptualization of social support while offering novel insights into the mechanics of adverse events within marital systems.

## 2 Methods

### 2.1 Participants

Participants were recruited through community service centers and senior activity organizations via convenience sampling, with inclusion criteria set at age ≥60 years. Because the core variable examined in this study was marital satisfaction, only currently married older adults were included. Individuals who were widowed, divorced, or never married were excluded after questionnaire completion. Inclusion criteria were: (1) aged ≥ 60 years; (2) able to complete the questionnaire independently or with minimal assistance. Exclusion criteria were: (1) serious hearing or speech impairments that prevented comprehension; (2) refusal to sign the informed-consent form. This study was approved by the Institutional Review Board of Qufu Normal University, and all participants provided written informed consent before completing the survey. The initial sample consisted of 500 older adults. Considering the common visual impairments among the elderly population, this study adopted an assisted data collection method. Specifically, volunteers who had undergone systematic training would read out the questionnaire content to the participants word for word, and then accurately record the participants’ verbal responses on the questionnaire. Rigorous quality control excluded 31 invalid responses based on predefined criteria: logical contradictions within responses; omission of critical items (with individual item non-response rates exceeding 15%); internally inconsistent multiple-choice selections; and evidence of non-substantive responding (e.g., identical scaling across items). Pre-defined data-quality criteria were applied before analysis: (1) Item response rate: missing items ÷ total items per scale; if any core scale (NLEs, UCLA, marital satisfaction, or partner support) had ≥ 15% missing, the whole questionnaire was discarded. (2) Completion time: recorded from survey opening to submission; questionnaires taking > 30 min were excluded. After applying the exclusion criteria, 469 usable questionnaires remained, representing 93.8% of the 500-person sample. Of the 469 participants, 241 identified as male (51.4%) and 228 as female (48.6%). Key demographic characteristics—including age distribution, highest level of educational attainment, and geographic region—are presented in Table 1.

**Table 1:** Description of sample feature distribution.

Variable	Option	Frequency	Percentage
Marital status	Married	424	90.4%
	Widowed	38	8.1%
	Never Married	7	1.5%
Gender	Male	241	51.4%
	Female	228	48.6%
Education level	Primary School	67	14.3%
	Junior High School	54	11.5%
	Senior High School/Vocational College/Diploma	172	36.7%
	Undergraduate	148	31.6%
	Graduate Program	28	6.0%
Age group	60–75	355	75.7%
	75–85	108	23.0%
	>85	6	1.3%
Annual income range	<¥20,000	89	19.0%
	¥20,000–¥50,000	131	27.9%
	>¥50,000	249	53.1%
Self-care ability	Fully Independent	418	89.1%
	Mild Disability	46	9.8%
	Moderate Disability	4	0.9%
	Severe Disability	1	0.2%
Number of chronic diseases	None	174	37.1%
	One	218	46.5%
	Two	53	11.3%
	Three or More	24	5.1%

### 2.2 Measures

#### 2.2.1 Negative life events assessment

Negative life events were measured using a module adapted from Professor Hu Zhi’s localized version of the Health Status and Associated Factors Survey for Older Adults (Hu et al., 2007). This instrument employs binary response options (yes/no) across seven categories of typical adverse life events (e.g., health deterioration, financial hardship, bereavement), with a total score range of 0–7. Specifically, these events include deterioration of health, financial hardship, widowhood, family conflicts, breakdown of social relationships, loss of close friends, and serious illness of family members. Each event is scored 1 if it occurred and 0 if not. Total scores range from 0 to 7, with higher scores indicating a greater number of negative life events experienced. The excellent internal consistency reliability observed in our sample (Cronbach’s *α* = 0.896) provides preliminary empirica support for the applicability of this localized scale among Chinese older adults.

#### 2.2.2 Loneliness measurement (UCLA-3)

Loneliness was assessed using the third edition of the UCLA Loneliness Scale (Russell, 1996), comprising 20 items rated on a 4-point Likert scale. Total scores reflect individual levels of loneliness. The scale contains 20 items, each rated on a 4-point frequency scale (1 = never, 2 = rarely, 3 = sometimes, 4 = often). Total scores range from 20 to 80, with higher scores reflecting stronger feelings of loneliness. As a globally validated instrument, its psychometric properties have been confirmed among samples of older Chinese adults (Xie et al., 2022). In this study, the scale demonstrated superior reliability (*α* = 0.955).

#### 2.2.3 Marital satisfaction scale

MS was evaluated using the subscale from the ENRICH Marital Quality Inventory developed by Fowers and Olson (1993), which systematically examines relationship quality through 10 dimensions. Total scores are calculated by summing items, with higher scores indicating greater satisfaction. The scale assesses marital quality across 10 dimensions, including communication, conflict resolution, shared activities, and emotional support. Each dimension is rated on a 5-point scale (1 = very dissatisfied, 5 = very satisfied). Total scores range from 10 to 50, with higher scores indicating greater marital satisfaction. The scale’s reliability and validity have been established in prior domestic studies (Li et al., 2022). Here, it achieved an *α* coefficient of 0.91, meeting psychometric standards.

#### 2.2.4 Partner support assessment

Partner support was measured using the Spousal Intimacy and Relational Responsiveness Scale (Dehle et al., 2001). The 25-item scale uses a 5-point frequency response scale (1 = never to 5 = always). The scale comprises 25 items rated on a 5-point frequency scale (1 = never, 2 = rarely, 3 = sometimes, 4 = often, 5 = always). Total scores range from 25 to 125, with higher scores indicating stronger perceived partner support. Empirical evidence has validated its applicability in spousal relationships (Stafford et al., 2019; Xiong et al., 2022). In this study, the scale showed excellent reliability (*α* = 0.948).

### 2.3 Data analysis

Psychometric properties (reliability, validity, and confirmatory factor analysis) were examined using *AMOS 24.0. SPSS 25.0*, integrated with the Process macro, was utilized for common method bias testing, descriptive statistics, correlation analyses, and mediation and moderation effect tests.

## 3 Results

### 3.1 Confirmatory factor analysis

Common method bias was evaluated using Harman’s single-factor test. Results indicated nine eigenvalues exceeding 1, with the largest factor accounting for 24.06% of variance (below the 40% threshold), suggesting no substantial common method variance. Subsequent confirmatory factor analysis (CFA) using *AMOS 24.0* yielded satisfactory model fit indices: *χ*^2^/df = 1.956 (<3), RMSEA = 0.045 (<0.05), CFI = 0.919 (>0.9), TLI = 0.913 (>0.9), and RFI = 0.837 (>0.8). These metrics collectively meet established criteria for structural equation models, demonstrating adequate fit between the theoretical model and observed data (Table 2).

**Table 2:** Model fitness test.

Index	Reference criteria	Observed value	Fit level
CMIN/DF	Excellent: 1–3; Good: 3–5	1.956	Excellent
RMSEA	Excellent: <0.05; Good: <0.08	0.045	Excellent
RFI	Excellent: >0.9; Good: >0.8	0.837	Good
CFI	Excellent: >0.9; Good: >0.8	0.919	Excellent
TLI	Excellent: >0.9; Good: >0.8	0.913	Excellent

### 3.2 Descriptive statistics and correlation analysis

Descriptive statistics (see Table 1) revealed that older adults reported a mean frequency of NLEs of *1.57* (*SD* = 0.39), corresponding to an estimated average of *1–2* events per participant. Loneliness scores were within the moderate-to-high range (*M* = 2.50, *SD* = 0.58), with the standard deviation indicating substantial interindividual variability. Both MS (*M* = 3.05, *SD* = 0.70) and partner support (*M* = 3.47, *SD* = 0.69) scores indicated generally high marital quality and strong partner support within the sample. However, notable heterogeneity was observed across individuals, particularly evident in the broader distribution of MS scores (Table 3).

**Table 3:** Descriptive statistics and correlation matrix of study variables.

Variable	*M* ± *SD*	1	2	3	*X*	*M*	*W*	*Y*
Self-care ability	1.12 ± 0.37	1						
Number of chronic diseases	1.84 ± 0.82	0.30^**^	1					
Annual income range	2.34 ± 0.78	−0.12^**^	0	1				
*X*	1.57 ± 0.39	0.19^**^	0.41^**^	−0.33^**^	1			
*M*	3.05 ± 0.70	−0.34^**^	−0.55^**^	0.30^**^	−0.52^**^	1		
*W*	3.47 ± 0.69	−0.41^**^	−0.62^**^	0.42^**^	−0.51^**^	0.65^**^	1	
*Y*	2.5 ± 0.58	0.34^**^	0.56^**^	−0.38^**^	0.49^**^	−0.67^**^	−0.69^**^	1

*indicates significance at the 0.05 level; **indicates significance at the 0.01 level; ***indicates significance at the 0.001 level. All values are rounded to two decimal places. Dummy variables include: self-care ability (1 = fully independent, 2 = mild disability, 3 = moderate disability, 4 = severe disability); number of chronic diseases (1 = none, 2 = one, 3 = two, 4 = three or more); annual income range (1 = <¥20,000, 2 = ¥20,000–¥50,000, 3 = > ¥50,000). *X*, negative life events; *M*, marital satisfaction; *W*, partner support; *Y*, loneliness. The same coding applies hereafter.

Pearson correlation analysis among study variables yielded several salient findings. NLEs demonstrated a significant positive association with loneliness (*r* = 0.48, *p* < 0.01), consistent with Hypothesis 1. Notably, NLEs exhibited a moderate negative correlation with MS (*r* = −0.52, *p* < 0.01), while MS showed a stronger negative correlation with loneliness (*r* = −0.67, *p* < 0.01). This pattern implies that marital quality may influence the relationship between life stressors and psychological well-being. Partner support exhibited two compelling significant associations: a strong positive correlation with MS (*r* = 0.65, *p* < 0.01) and a strong negative correlation with loneliness (*r =* −0.69, *p* < 0.01). These findings lend empirical support to the protective role of marital relationships in later life. The identified correlation structure provides a robust basis for subsequent structural equation modeling to explore potential relationships among these variables.

### 3.3 Mediation analysis

To examine the mediating role of MS between NLEs and loneliness, mediation analysis was performed using Model 4 in *PROCESS macro* v4.0. Controlling for annual income level, self-care ability, and chronic disease count, the analysis treated NLEs as the independent variable, MS as the mediator, and loneliness as the dependent variable, utilizing 5,000 bootstrap samples. As shown in Table 4, results confirmed significant mediation effects: MS yielded an indirect effect of 0.428, while the total effect was 0.729, representing 58.71% of the total effect explained through mediation. The 95% bias-corrected confidence interval for the indirect effect [0.337, 0.532] did not include zero, further validating its statistical significance. These findings support the viability and robustness of MS as a mediator, thereby supporting Hypothesis 2.

**Table 4:** Testing of the mediating effect of MS.

Path	Effect	Proportion	Bootstrap SE	Bootstrap 95%CI	
				Lower 95% CI	Upper 95% CI
*X* → *Y*	Total effect				
	0.729		0.108		
*X* → *Y*	Direct effect				
	0.301	41.29%	0.058	0.187	0.415
*X* → *M* → *Y*	Indirect effect				
	0.428	58.71%	0.05	0.337	0.532

*indicates significance at the 0.05 level; **indicates significance at the 0.01 level; ***indicates significance at the 0.001 level. All values are reported to two decimal places. *X*, negative life events; *M*, marital satisfaction; *Y*, loneliness.

### 3.4 Moderated mediation analysis

Hierarchical multiple regression analyses were conducted using *SPSS Macro Model 8* to examine the moderating role of partner support in the association between NLEs (independent variable) and loneliness (dependent variable). We focused on the pathway from NLEs (X) to loneliness (Y), specifying MS (M) as a mediator and partner support (W) as a moderator (detailed results in Table 5).

**Table 5:** Testing of moderated mediation effects.

Variable	*M*			*Y* (overall moderating effect)			*Y*		
	*β*	*t*	95%CI	*β*	*t*	95%CI	*β*	*t*	95%CI
*X*	−0.81	−8.44***	−1.00, −0.62	0.38	0.10***	0.18, 0.57	0.61	6.33^***^	0.42, 0.80
*M*				−0.41	0.06***	−0.54, −0.28			
*W*	−0.61	−10.88***	0.50, 0.72	−0.58	0.06***	−0.70, −0.46	−0.75	−13.44^***^	−0.86, −0.64
X*W	−0.31	8.77***	0.24, 0.38	−0.13	0.37***	−0.20, -0.60	−0.22	−6.19***	−0.29, −0.15
*R* ^2^	0.54			0 0.58				0.54	
*F*	184.96			158.84				132.60	

*p < 0.01, **p < 0.001. All values are reported to two decimal places. *X* = NLEs; *M*, marital satisfaction; *Y*, loneliness; *W*, partner support.

In the loneliness (Y) model, NLEs (X) significantly positively predicted loneliness (*β* = 0.61, *t* = 6.33, *p* < 0.001), while partner support (W) exerted a significant negative effect (β = −0.75, *t* = −13.44, *p* < 0.001). Notably, the interaction term (X × W) significantly negatively predicted loneliness (*β* = −0.22, *t* = −6.19, *p* < 0.001), indicating that higher partner support attenuates the positive association between NLEs and loneliness. The model explained 54% of variance (*R*^2^ = 0.54, *F* = 132.60, *p* < 0.001), supporting Hypothesis 3.

For the MS (M) model, NLEs (X) exerted a significant negative effect (*β* = −0.81, *t* = −8.44, *p* < 0.001), and partner support (W) similarly showed a significant negative effect (*β* = −0.61, *t* = −10.88, *p* < 0.001). The interaction term (X × W) significantly positively predicted MS (*β* = −0.31, *t* = 8.77, *p* < 0.001), suggesting that partner support negatively moderates the link between NLEs and MS. This model accounted for 54% of variance (*R*^2^ = 0.54, *F* = 184.96, *p* < 0.001).

**Figure 1:**
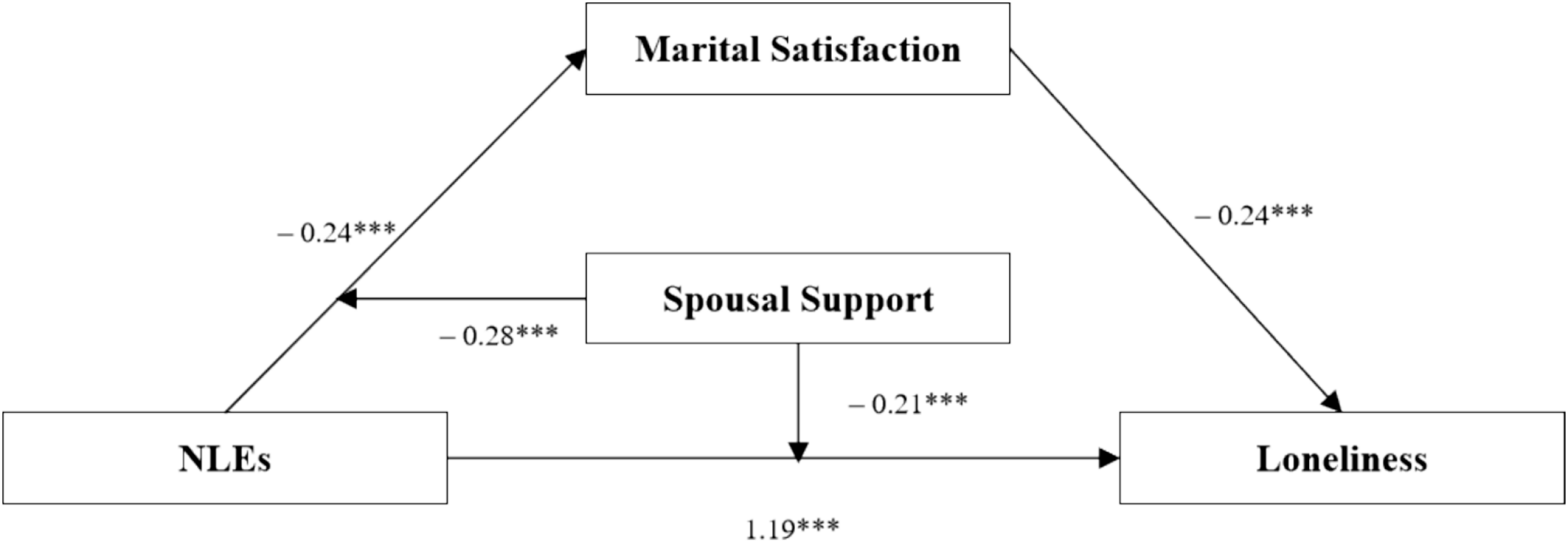
Path coefficient diagram.

In the full moderated mediation model controlling for self-care ability, chronic disease count, and annual income, NLEs (X) remained a significant positive predictor of loneliness (*β* = 0.38, *t* = 10.10, *p* < 0.001), while MS (M) significantly negatively predicted loneliness (*β* = −0.41, *t* = −6.06, *p* < 0.001). The interaction term (X × W) continued to significantly predict loneliness (*β* = −0.13, *t* = 3.7, *p* < 0.001), confirming partner support’s moderating role in the first stage of the mediation pathway. This final model explained 58% of variance (*R*^2^ = 0.58, *F* = 158.84, *p* < 0.001), providing strong empirical support for Hypothesis 4 (Figure 1).

## 4 Discussion

Grounded in the socioecological model and stress response theory, this study focused on the complex interplay among NLEs, MS, partner support, and loneliness in older adults. The findings not only corroborate the direct predictive effect of NLEs on loneliness but also elucidate the mediating role of MS in this relationship. Furthermore, they highlight the critical moderating role of partner support along the hypothesized relationship. Subsequent sections will provide a detailed interpretation of these three core empirical discoveries.

### 4.1 Negative life events and loneliness in older adults

The primary finding of this study establishes that NLEs are significantly and positively associated with loneliness among older adults. This result aligns strongly with extensive prior research, reaffirming the status of NLEs as a critical risk factor for psychological distress—particularly loneliness—in later life (Aartsen and Jylhä, 2011; Li et al., 2024; Wei et al., 2022). Additionally, this study elucidates the mechanisms through which negative life events are related to loneliness and, from a marital perspective, examines the pivotal impact of spousal support—an essential component of social support—on loneliness. A key innovation of our study is the introduction of marital satisfaction and partner support, moving beyond the scope of Haehner et al. (2024b), which centered solely on the perception of major life events and psychological adaptation. In doing so, it extends the boundaries of Haehner et al. (2024a) and broadens the research frontier on how partner support influences the pathways to loneliness. From a socioecological perspective, such events (e.g., spousal bereavement, chronic illness diagnosis, intergenerational separation) represent significant disruptions within the microsystem (Bronfenbrenner, 2000), directly related to shrinkage or fragmentation of social networks and resulting in substantive losses of social connection resources (Switsers et al., 2021; Tang et al., 2024). This disintegration of objective social relationship structures forms the foundational underlying loneliness emergence (Aartsen and Jylhä, 2011; Li et al., 2024). Through the PROCESS regression framework, our study precisely quantifies this direct effect pathway, providing robust empirical evidence for understanding external triggers of late-life loneliness. From an ecological perspective, marital satisfaction and spousal support emerge as core resources within the family microsystem, highlighting the pivotal role of the family context in older adults’ psychological adaptation.

More importantly, the impact mechanism extends beyond mere social disconnection to encompass systemic depletion of psychological resources among older adults (Switsers et al., 2021; Wei et al., 2022). According to the stress process model (Pearlin et al., 1981), NLEs can lead to a sense of helplessness and loss of control, which undermines the emotional security provided by a high-quality marriage. This disruption in marital satisfaction can then amplify feelings of loneliness as the couple’s ability to provide mutual support and reassurance is compromised (Fincham and Bradbury, 1993). According to stress response theory, aging individuals possess inherently limited coping resources due to physiological decline and reduced psychological elasticity (Liao et al., 2022). When stressor loads exceed individual thresholds, sustained pressure progressively exhausts cognitive and emotional reserves, precipitating pronounced feelings of helplessness, loss of control, and diminished self-worth (Wei et al., 2022). Such psychopathological processes are associated with voluntary social withdrawal and fosters negative cognitive biases toward existing relationships, thereby amplifying perceived isolation (Li et al., 2024).

### 4.2 The mediating role of marital satisfaction

The second core finding reveals that MS partially mediates the relationship between NLEs and loneliness in older adults. This discovery holds theoretical significance by illuminating the mechanism through which external stressors related to internalized loneliness. As the cornerstone of elderly social support networks, marital quality functions as a protective barrier against external risks and preserves psychological well-being (Murray et al., 2013). When NLEs occur, attendant stress does not dissipate but permeates individuals’ primary relational domains (Dion et al., 2023; Nguyen et al., 2021). This “stress spillover” effect substantially erodes marital quality, manifested as increased spousal conflict, diminished communication quality, and mutual withdrawal—ultimately reducing MS (Dion et al., 2023; Nguyen et al., 2021). Our mediation analysis empirically supports this theoretical framework.

The exacerbation of loneliness via reduced MS stems from impaired “psychological safe haven” functionality within marriage (Murray et al., 2013; Tang et al., 2024). High-quality marriages provide stable emotional sustenance and fulfill needs for understanding and belonging; conversely, declining MS weakens this emotional tether, resulting in older adults experiencing profound intramarital loneliness—characterized by emotional disconnection despite marital coresidence (Aguilar-Raab et al., 2022; Tang et al., 2024). Such emotional isolation is suggested to be more detrimental than social loneliness associated with living alone. Our identification of partial mediation indicates that while NLEs may directly influence loneliness through other pathways (e.g., reduced social engagement), their amplification of loneliness via marital relationship disruption constitutes a clinically significant mechanism (Aguilar-Raab et al., 2022). This underscores the need to assess older adults within familial microsystems. Thus, evaluating relationship quality is indispensable for comprehensive mental health screening.

### 4.3 The moderating effect of partner support

The theoretical novelty of this study lies in its preliminarily identify the moderating role of partner support under specific contexts and their profound implications for psychological health transmission pathways. Its regulatory influence extends beyond direct intervention in the association between NLEs and loneliness, critically manifesting bidirectional moderation at the antecedent stage of the mediation pathway—specifically, during the transformation of NLEs into MS.

Regarding direct effects, partner support’s buffering function against adversity corroborates the classical stress-buffering model (Lam, 2024). From the perspective of social support theory (Cohen and Wills, 1985), spousal support enhances individuals’ ability to cope with negative life events by providing emotional comfort and practical assistance, thereby mitigating the impact of these events on loneliness. Additionally, according to family systems theory (Minuchin, 2018), spousal support plays a stabilizing and coordinating role within the family system, helping couples maintain relational harmony and balance when facing stress, which further alleviates loneliness. When individuals receive sustained emotional solace, information provision, and practical assistance from spouses, these social resources effectively mitigate the immediate psychological impact of stressors, sustain positive self-conceptualization, and preserve perceived control over one’s environment—thereby interrupting the trajectory toward loneliness (Wilson et al., 2021).

Particularly noteworthy is the distinctive moderation pattern observed in the early segment of the mediation pathway. Empirical evidence demonstrates that under conditions of low partner support, NLEs significantly amplify the decline in MS (Aguilar-Raab et al., 2022). This phenomenon vividly illustrates the classic crisis amplification effect: When partners lack collaborative coping capacities, exposure to external stressors not only fails to generate unified resilience but instead plunges them into mutual recrimination spirals, precipitating accelerated deterioration of marital quality (Panahi et al., 2018), ultimately perpetuating a vicious cycle of stress → relationship erosion → loneliness.

The most theoretically innovative finding emerges from high-partner-support contexts, within which NLEs paradoxically predict increased MS—transcending traditional buffering model explanations and aligning with constructs of “post-stress growth” and “relationship resilience” (Zhao et al., 2022). Within high-quality marriages, partners frame themselves as shared destiny actors, transforming external challenges into opportunities for relational deepening through coordinated coping strategies (Koranyi et al., 2017). The collective struggle itself becomes an affective crucible fostering mutual understanding, fortifying trust bonds, and cultivating gratitude. This transformative mechanism poignantly illustrates partner support’s dual nature: serving simultaneously as both a protective shield against adversity and a catalytic agent for relational transcendence—successfully converting potential destructive forces into relationship-strengthening momentum.

## 5 Conclusion

This study systematically elucidates the multidimensional pathways and complex mechanisms by which NLEs influence loneliness in older adults. Findings indicate that such events are not only directly associated with loneliness but also indirectly associated through their link with lower MS, a cornerstone of social support. Crucially, partner support emerges as a pivotal moderator: it both directly cushions individuals against psychological impacts of stress and fundamentally reframes the meaning of stressful events within marital relationships. Under low-support conditions, stress functions as a “corrosive agent” for marital bonds; conversely, high-support conditions transform stress into a “relationship binder.” These insights advance theoretical understanding of late-life mental health while carrying significant clinical implications. Future interventions should transcend traditional individual-focused approaches, adopting couple-centered family systems strategies that fortify supportive behaviors and collaborative coping skills within partnerships. Such systemic enhancement providing a robust defense against life’s adversities and serves to prevent and alleviate late-life loneliness.

The present study has several limitations. First, due to its cross-sectional design, the temporal sequence among negative life events, marital satisfaction, partner support, and loneliness could not be determined. Additionally, the potential influence of reverse causation and unmeasured confounding variables on the results cannot be entirely ruled out. Second, at the measurement level, the use of the unidimensional UCLA Loneliness Scale prevented effective differentiation between the social and emotional subtypes of loneliness. To address these limitations, future research should consider two primary directions: (a) employ multidimensional assessment tools capable of simultaneously evaluating social and emotional loneliness to elucidate the differential mechanisms by which negative life events affect distinct dimensions of loneliness; (b) conduct rigorous experimental studies through systematic manipulation of situational stressors and implementation of randomized intervention strategies, thereby validating the potential causal pathways among these variables.

## Data Availability

The raw data supporting the conclusions of this article will be made available by the authors, without undue reservation.
